# Neuromapping of the Capsuloligamentous Knee Joint Structures

**DOI:** 10.1016/j.asmr.2020.12.009

**Published:** 2021-02-23

**Authors:** Andreas Martin Seitz, Miriam Murrmann, Anita Ignatius, Lutz Dürselen, Benedikt Friemert, Falk von Lübken

**Affiliations:** a Institute of Orthopedic Research and Biomechanics Institute of Orthopedic Research and Biomechanics Center for Trauma Research Ulm, Ulm University Medical Center, Ulm, Germany; b Department of Trauma and Orthopedic Surgery, Hospital of the Federal Armed Forces Ulm, Ulm, Germany

## Abstract

**Purpose:**

To investigate neuromuscular electromyographic response of the of the upper and lower leg muscles after the application of an intraoperative, isolated mechanical stimulus of the capsuloligamentous structures, including the anterior (ACL) and posterior cruciate ligaments (PCL), lateral (LM) and medial menisci (MM), plica mediopatellaris (PM), and Hoffa’s fat pat (HFP).

**Methods:**

The electromyographic response of the upper and lower leg muscles (M. rectus femoris; M. vastus medialis; M. semitendinosus; M. biceps femoris; M. gastrocnemius lateralis) of 15 male patients were measured after an isolated mechanical stimulus of the capsuloligamentous structures during an arthroscopic intervention using a customized intraoperative setup. Target parameters were the short (SLR; <30 milliseconds) and medium latency responses (MLR; >30 milliseconds) after the mechanically-induced trigger.

**Results:**

The ACL, PCL, LM, and MM displayed high interindividual reproducibility of >76%. The MM was the only structure indicating both an SLR and MLR for all muscles. Although signals could be detected, there was no reproducibility in electromyographic signal activation for the HFP. The most rapid MLR was observed for the PM (quadriceps: 37 milliseconds).

**Conclusions:**

Each stimulated structure displayed an individual MLR response, which allowed us to create neuromapping combining the anatomical and quantitative representations of the individual muscular activation patterns after isolated mechanical stimulation of the capsuloligamentous knee joint structures, corroborating our hypothesis.

**Level of Evidence:**

Diagnostic - Level II.

With an incidence of 37%, the knee is one of the most frequently injured joints during sports activities.^1^ Injuries of the capsuloligamentous structures of the knee are one of the most common diagnoses in everyday clinical practice, showing a high incidence both after sports injuries and trauma and in the context of degenerative diseases.^1^^,^^2^ The knee joint can be injured by a direct impact in contact sports, including football and soccer, or by a rotational movement without the influence of an external opponent, for example, during skiing activities. The directly associated treatment costs are themselves of socioeconomic importance, and the majority of patients affected are in the workforce.^3^ The associated indirect follow-up costs caused by loss of working hours and early arthrosis are difficult to quantify, but they are assumed to be at least 4 times greater than the direct costs.^3^

Flexion–valgus external rotation trauma of the knee joint is one of the most frequent noncontact injury mechanisms^2^^,^^4^ and can lead to a severe combination trauma of the capsuloligamentous knee joint structures, which is commonly associated with a loss of proprioception and knee joint stability. The resulting loss of proprioception and instability can be caused mechanically as well as neuromuscularly. The latter is also known as a functional instability and can be explained by a disturbance of the sensorimotor regulation of the knee joint. The hereby damaged mechanoreceptors lose their function to adequately provide an afferent stimulus. Loss of this reflex leads, by a loss of muscle coordination, to a loss of protective joint function. The clinical equivalent to this are giving-way episodes, resulting in a continuously increasing instability symptomatology,^5^ which can lead in the long term to micro-and macrocartilage lesions and finally to premature osteoarthritis of the knee.

In addition to their function as mechanical stabilizers, the anterior (ACL) and posterior (PCL) cruciate ligaments play an important role within the neuromuscular feedback mechanism.^6^, ^7^, ^8^, ^9^, ^10^, ^11^, ^12^, ^13^, ^14^ Clinically, there are 2 different types of patients presenting with ACL concerns: coper and noncopers. In contrast to the patients who are copers, that is, they feel subjectively stable and are able to largely resume pretraumatic activity, patients who are noncopers exhibit considerable subjective functional instability without any indication of a severe mechanical instability, leading to the so-called giving-way symptom (GWS).^15^, ^16^, ^17^ Chmielewski et al.^15^ described functional joint stability as the ability of the knee joint to remain stable during everyday physical movements without displaying a GWS. The GWS can be clinically defined as a disturbed reflex control loop, creating an undesired muscular relaxation around the knee, which leads to an insufficient stabilization and thus to a subluxation of the knee. In human and animal experiments, a reflex control loop was identified between the ACL and the hamstring muscles.^8^^,^^9^^,^^13^^,^^14^^,^^18^, ^19^, ^20^, ^21^ It is established that the different neuroreceptors in the tissues of the knee joint^22^^,^^23^ contribute to a different extent to the proprioceptive function of the healthy and degenerated joint.^24^^,^^25^ Therefore, it is possible that other capsular and ligamentous structures also contribute to the functional stability of the knee by similar reflex control loops.^26^^,^^27^

Because of the severity of the injury, the limited therapeutic options, and the according risk factors that are very important for the long-term outcome, the adequate treatment of capsuloligamentous injuries of the knee joint is of paramount importance. The primary aim of this study was to test whether knee joint structures other than the ACL and PCL contribute to knee joint proprioception. One of the major issues of recent detection methods is that when an external mechanical stimulus is triggered, multiple receptors of the knee joint can be activated, resulting in a specific neuromuscular activation. However, it is unknown which single anatomical structure causes the observed muscular response.^28^^,^^29^ The purpose of this study was to investigate neuromuscular electromyographic response of the of the upper and lower leg muscles after the application of an intraoperative, isolated mechanical stimulus of the capsuloligamentous structures, including the ACL, PCL, lateral meniscus (LM), medial meniscus (MM), plica mediopatellaris (PM), and Hoffa’s fat pad (HFP). We hypothesized that an isolated mechanical stimulus not only of the ACL or PCL but also of the menisci, PM, or the HFP creates a distinct neuromuscular electromyographic (EMG) response of the upper and lower leg muscles.

## Methods

### Patients

Following institutional review board approval (Ulm University, no. 91/11) and the patients’ written informed consent, the ACL, PCL, LM and MM, PM, and HFP of 17 male patients (Table 1) with no history of neurologic disorders were investigated during different arthroscopic interventions. Because of interference frequencies of the EMG signals in 1 patient (patient 1) and poor recording of the mechanical trigger signal in another patient (patient 7), we were able to evaluate measurements from 15 patients. All patients underwent general anesthesia (total intravenous anesthesia) for the surgical procedure with 50 to 150 μg/kg/min propofol. The use of such low doses is unlikely to affect the monosynaptic reflex arc.^30^ No further relaxants or local anesthetics, spinal anesthesia, or catheters were used before or during the measurements.

**Table 1:** Demographic Patient Data

No.	Age, y	Height, cm	Weight, kg	Indication
1∗	19	183	72	ARY, microfracture
2	20	185	95	Diagnostic ARY
3	26	176	76	ARY, MPFL Recon
4	51	175	80	Diagnostic ARY
5	21	193	120	ARY, ACL Recon: BTB
6	34	186	95	ARY, ACL Recon: BTB
7^#^	44	193	82	Diagnostic ARY
8	22	176	75	ARY, ACL Recon: STG
9	27	172	80	ARY, loose body resection
10	48	175	88	ARY, ACL Recon: STG
11	33	186	92	ARY, HTO
12	21	169	73	ARY, ACL Recon: STG
13	22	190	90	ARY, Drill hole filling
14	31	189	103	ARY, ACL Recon: STG
15	23	178	72	Diagnostic ARY
16	49	175	75	Diagnostic ARY
17	20	176	86	Diagnostic ARY
Mean	29.9 ± 11.1	180.1 ± 7.4	86.7 ± 13.2	–

NOTE. The last row summarizes the mean ± standard deviation values.

ACL, anterior cruciate ligament; ARY, arthroscopy; BTB, bone to bone graft; HTO, high tibial osteotomy; MPFL, medial patellofemoral ligament; Recon, reconstruction; STG, semitendinosus graft.

∗ Patients were excluded due to technical issues.

### Measurement Setup

**Fig 1:**
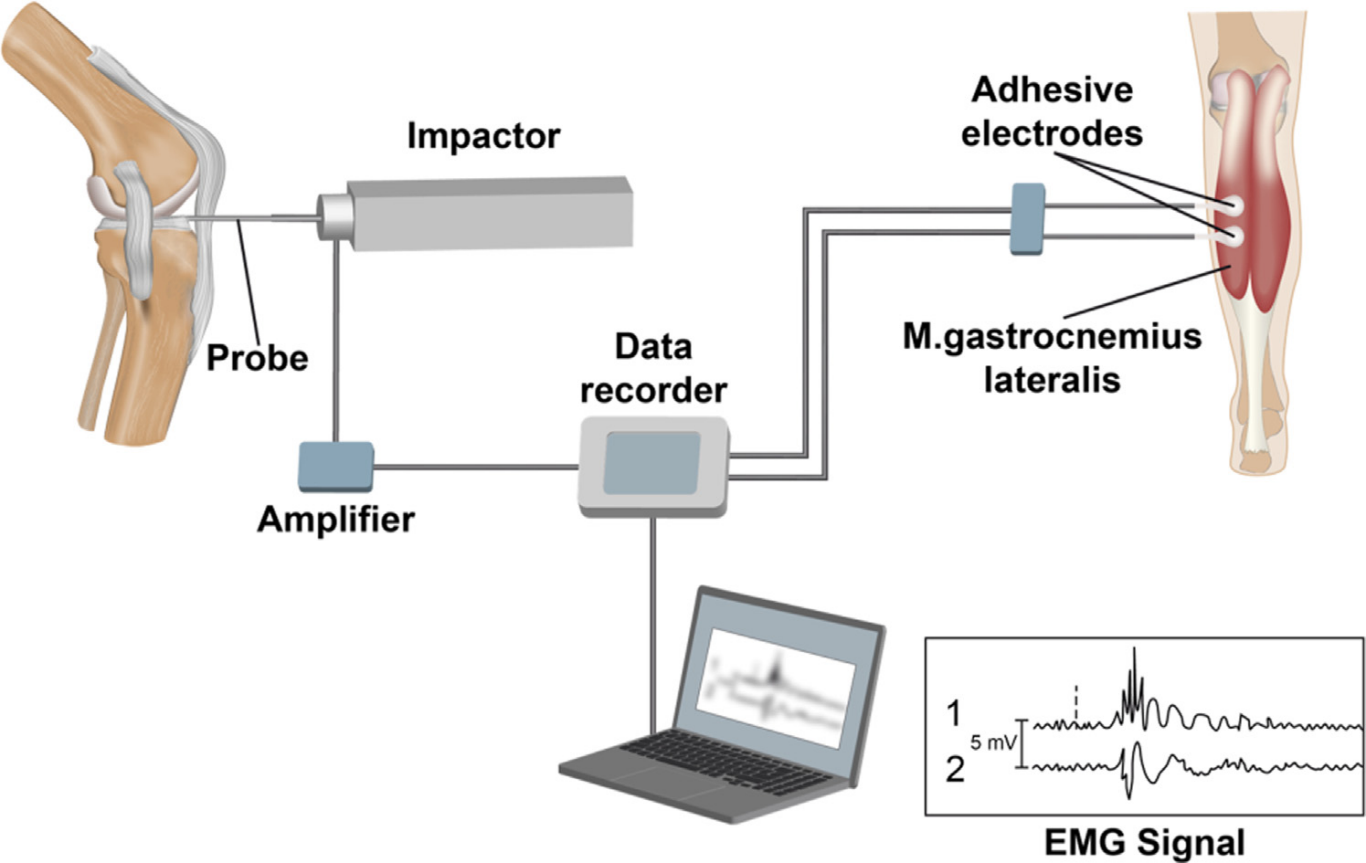
Schematic drawing of the test setup to measure the latency time between the isolated mechanical stimulation of the capsuloligamentous structure via the impactor and the increase of the electromyographic potential of the knee-spanning muscles (here: M. gastrocnemius).

**Fig 2:**
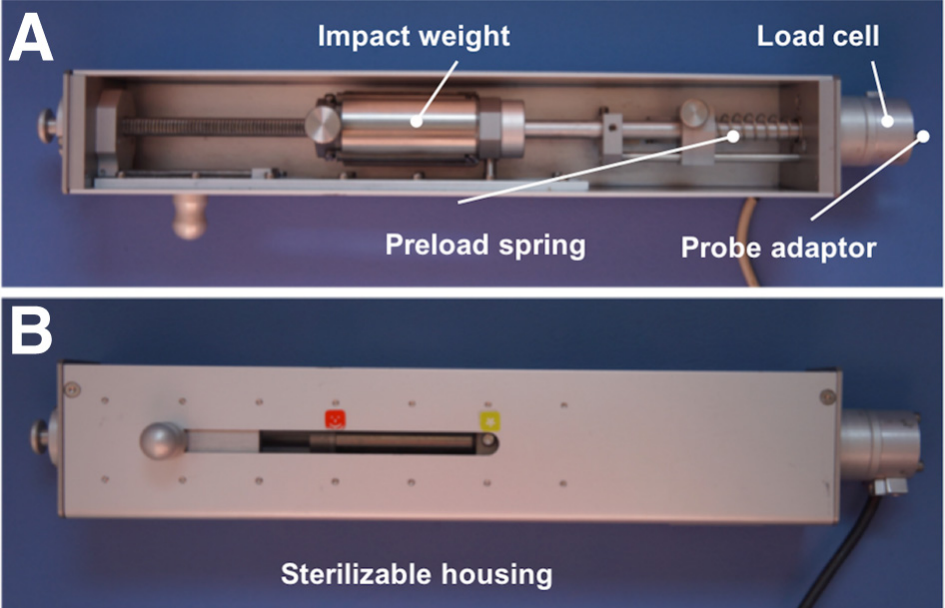
(A) Impactor allowing for an isolated mechanical stimulation of the capsuloligamentous knee joint structures (anterior and posterior cruciate ligament, lateral and medial meniscus, joint capsule, Hoffa’s fat pad). An arthroscopic hook probe can be directly attached to the load cell (8426-500 N, burster präzisionsmesstechnik GmbH, Germany) using the probe adaptor. (B) A defined force of 15 N (red position) or 40 N (yellow position) can be applied by tensioning the preload spring together with the impact weight to the respective position.

A novel customized muscle reflex measurement setup (Fig 1) was developed to allow for an isolated mechanical stimulation of the capsuloligamentous structures. Hereby, a commercially available arthroscopic hook probe (Richard Wolf GmbH, Knittlingen, Germany) with a modified rounded tip that was directly connected to an impactor allowed for the application of isolated subcritical stimuli of 15-N tensile force to the PM and HCP and 40 N to the ACL, PCL, LM, and MM (Fig 2). The impactor’s force signal was amplified and connected to an input box (USB Input box; Biovision, Wehrheim, Germany). The input box, which was connected via USB to the measurement computer, was also able to handle and process the EMG. The raw neuromuscular EMG data of the thigh and shank muscles (M. vastus medialis, M. rectus femoris, M. semitendinosus, M. biceps femoris, M. gastrocnemius lateralis) were collected at a sampling rate of 3 kHz, 1000× preamplified, and recorded with a commercially available evaluation software (Daisy Lab; Biovision). Electrode placement was conducted in accordance to the SENIAM guidelines^31^ using 2 adhesive electrodes (Kendall; Covidien, Dublin, Ireland) for each muscle head, while the reference electrode was positioned at the iliac spine. To achieve an optimal electrode contact with low skin impedance, the skin was shaved and cleaned with alcohol. The EMG signals were band-pass filtered (10-500 Hz) with an eighth-order Butterworth filter to eliminate artifacts. Validation and repeatability of linear envelope EMG measurements were ensured during pretests.

### In Vitro: Tensile Test

**Fig 3:**
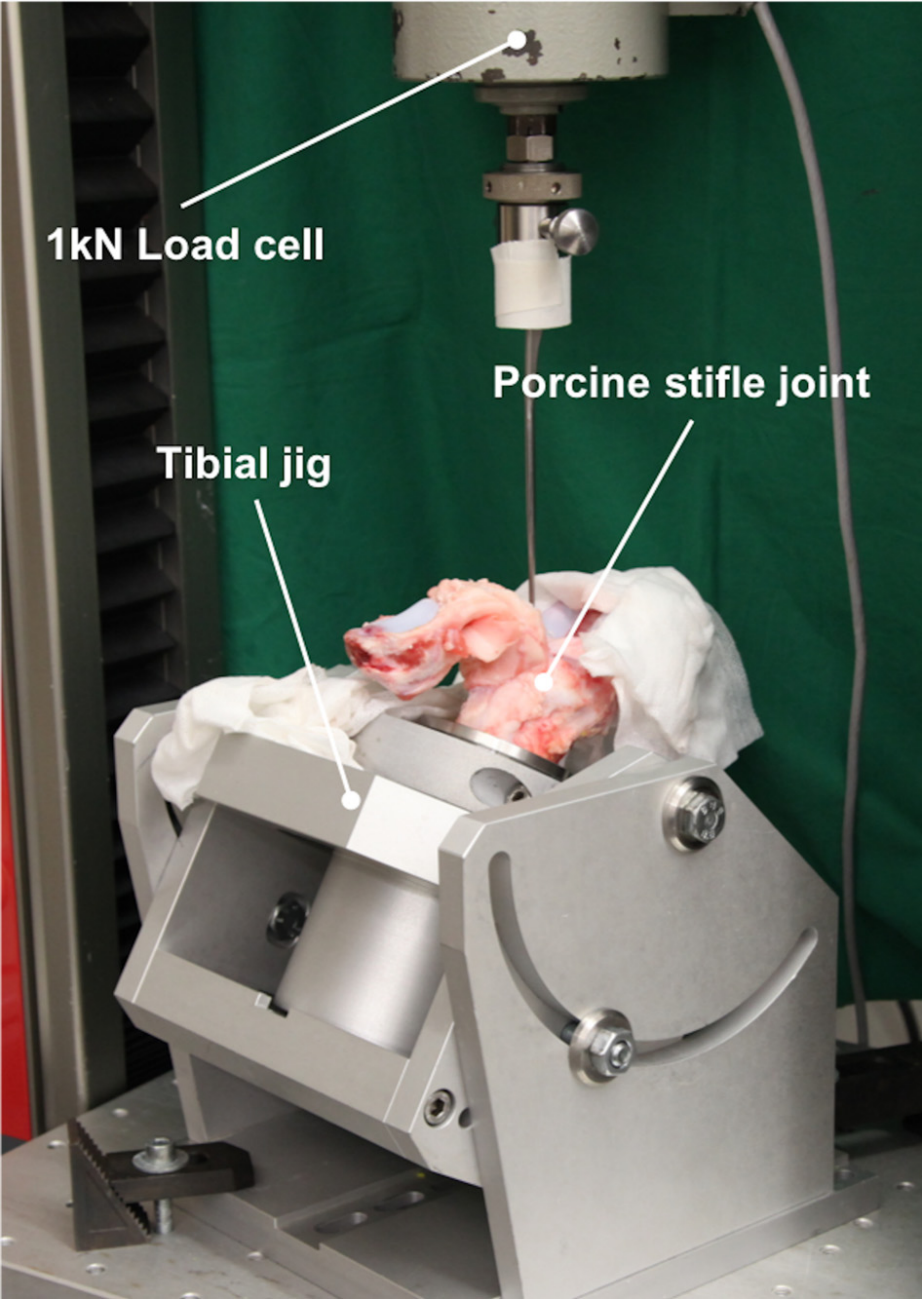
Overview of the tensile test setup with the porcine stifle joint aligned for testing of the lateral meniscus.

Pretests were conducted to prevent any injury to the capsuloligamentous structures during the in vivo measurements. Therefore, 6 intact cadaveric porcine stifle joints, obtained from a local butcher, were tested using a customized setup in a standard material testing machine (Fig 3; Z010; Zwick GmbH, Ulm, Germany). The porcine joints were potted in steel cylinders using polymethylmethacrylate (Technovit 3040; Kulzer GmbH, Hanau, Germany) and mounted in a tibial jig, which allowed free placement in 5 degrees of freedom. Subsequently, an arthroscopic hook, which was connected to a 1-kN load cell, applied maximum tensile loads of 50 N to the HFP and PM and 500 N to all other capsuloligamentous structures at a loading rate of 100 mm/min. During the measurements, the force elongation curves were recorded and the rupture force and the force at the transition from the toe to the linear region (Table 2), reflecting the typical behavior of soft tissues,^32^ were determined. This transition point was defined as the threshold for subcritical loads. Subsequently, we defined subcritical loads far below the failure loads of 40 N for the ACL, PCL, LM and MM and of 15 N for the HFP and PM, which were then used during the intraoperative mechanical stimulation.

**Table 2:** Tensile Forces, in N, of the Pretests

Structure	F_toe_, N	± SD, N	F_max_, N	± SD, N
ACL	232.8	48.3	>500	–
PCL	232.3	35.2	>500	–
LM	163.8	130.7	294.0	167.9
MM	146.3	122.5	332.0	60.4
HFP	16.0	11.4	26.0	12.3
PM	14.8	9.7	47.3	17.8

NOTE. The transition force (F_toe_) is determined at the transition from the toe to the linear region of the force-elongation curves and the rupture force (F_max_) represents the maximum measured force. The ACL and PCL did not fail at the maximum applied load of 500 N.

ACL, anterior cruciate ligament; HFP, Hoffa’s fat pad; LM, lateral meniscus; MM, medial meniscus; PCL, posterior cruciate ligament; PM, plica mediopatellaris; SD, standard deviation.

### In Vivo Measurements

The patients were positioned for arthroscopic knee surgery on the operating table and subsequently all electrodes were placed as described above and covered with sterile tape. A tourniquet (Tourniquet 5000; VBM Medizintechnik, Sulz am Neckar, Germany) was placed around the thigh and inflated for the surgical procedures after the measurements. Once the leg was cleaned and draped, a diagnostic arthroscopy was performed using an anterolateral approach. The sterile hook was inserted via the anteromedial approach. The stimuli of the ACL, PCL, and MM were conducted with the hook directly attached. For the stimulation of the LM, the approaches were changed, with insertion of the optics to the medial portal and the hook to the anterolateral portal. By contrast, the stimuli of the PM and HFP were performed using a surgical clamp that provided a larger application surface to which the hook was directly interlocked. The intraoperative measurements were repeated three times, while the tibia was fixed by means of fixation tape to prevent anterior tibial displacement. Following completion of the measurements, the indicated surgical procedure was performed. The target value of the measurements was the latency time between the isolated mechanical stimulation of the capsuloligamentous structures and the increase of the EMG potential of the knee-spanning muscles. Latencies <30 milliseconds are defined as short latency responses (SLR, monosynaptic tendon jerk reflexes), whereas latencies ≥30 milliseconds are defined as medium latency responses (MLR, polysynaptic spinal reflexes).^28^ A previously described algorithm was used to differentiate between the end of the SLR and the beginning of the MLR.^28^

### Statistical Analysis

Following a priori sample calculation (G∗Power 3.1^33^: α = 0.05, β = 0.2, dz = 2.08; n = 5), the mean latency response (SLR, MLR) of each capsuloligamentous structure was evaluated.

## Results

**Fig 4:**
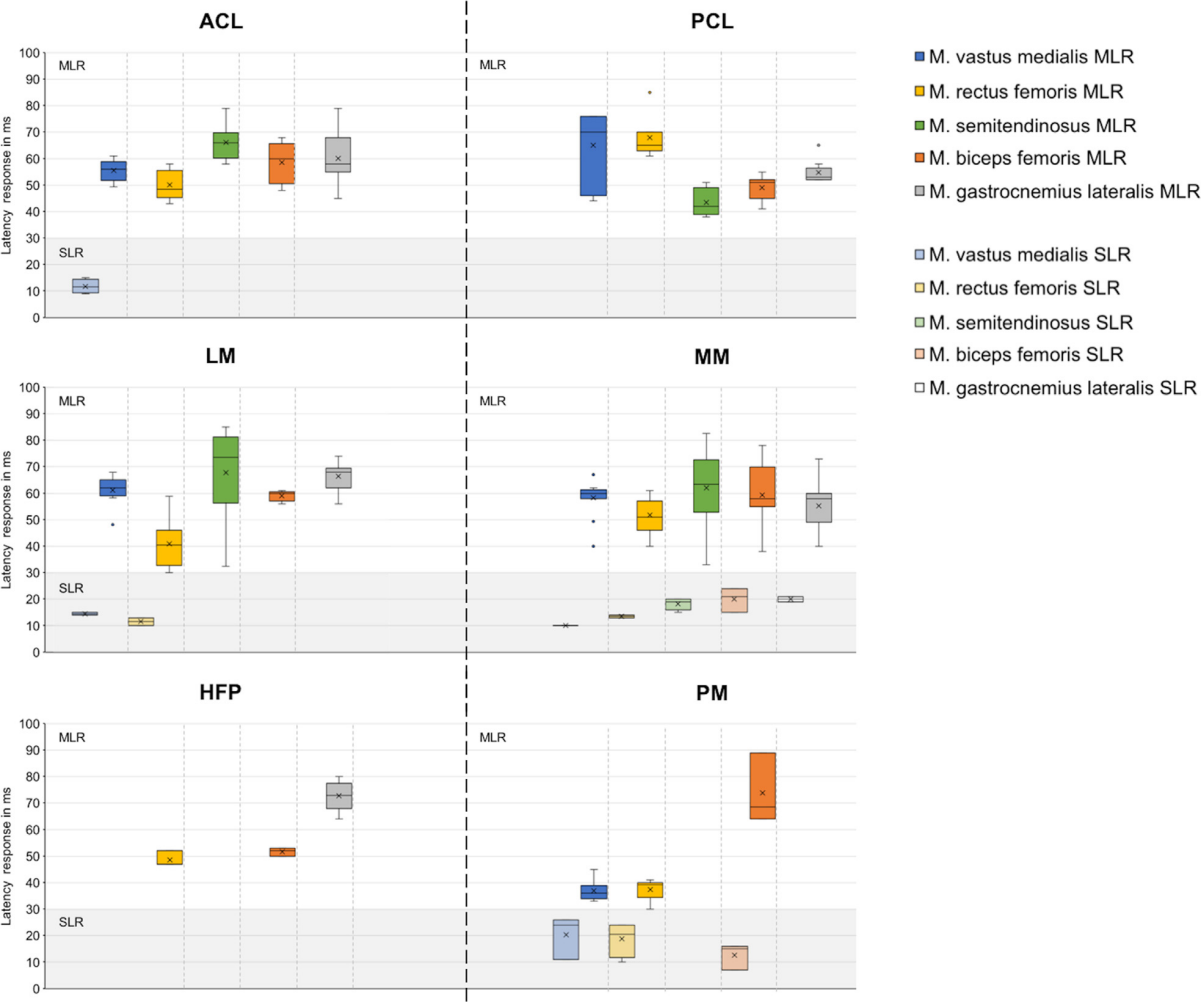
Mean (x), median, minimum, maximum, and 50% values for the SLR and MLR, in milliseconds, of 5 knee-spanning muscles (M. vastus medialis, M. rectus femoris, M. semitendinosus, M. biceps femoris, and M. gastrocnemius lateralis) after isolated mechanical stimulation of the ACL (n = 15 measurements), PCL (n = 12), LM (n = 21), MM (n = 24), HFP (n = 18), and PM (n = 18). The gray area indicates the time range of the SLR (t < 30 ms) and the white area indicates the time range of the MLR (t ≥ 30 ms) of the muscles. (ACL, anterior cruciate ligament; HFP, Hoffa’s fat pad; LM, lateral meniscus; MLR, medium latency response; MM, medial meniscus; PCL, posterior cruciate ligament; PM, plica mediopatellaris; SLR, short latency response.)

The mechanical stimulation of the ACL led to reproducible (n = 15; 98.3%) muscle response characteristics of all 5 muscles, which indicated an MLR ranging between 43 and 79 milliseconds (Fig 4). Regarding the SLR, only the M. vastus medialis displayed a reproducible response between 9 and 15 milliseconds.

With respect to the quadriceps and hamstring muscles, the isolated ACL and PCL stimulation caused a contrary reflex response. Following PCL stimulation (n = 12; 100% MLR reproducibility), the primary reflex response of the hamstrings occurred after 40 to 50 milliseconds with a subsequent quadriceps response. For the ACL stimulation the MLR response was vice versa.

Both for the LM and the MM the first MLR was detected for the M. rectus femoris (40-60 milliseconds) followed by the M. biceps femoris. Overall, both menisci displayed qualitatively similar EMG responses, except for the M. gastrocnemius lateralis. In contrast to the hamstring muscles, the quadriceps muscles indicated an SLR after the isolated stimulation of the LM (range 10-15 milliseconds). Differences were detected in the activation profile of the M. rectus femoris (n = 21; 94.6% reproducibility), whereas all other muscles indicated a high interindividual reproducibility. The MM was the only capsuloligamentous structure that indicated both an SLR (range: 10-24 milliseconds) and an MLR (range: 33-83 milliseconds) for all 5 muscles with a good reproducibility (n = 24; 76.2%).

Although some signals could be detected for the HFP, there was no reproducibility in the EMG signal (n = 18; 14.6%). The PM displayed the most rapid MLR at the quadriceps muscles (37 milliseconds) of all investigated structures. The response of the hamstring muscles was 30 to 40 milliseconds later. In general, the reproducibility of the muscle activation was low (n = 18; 30.6%).

**Fig 5:**
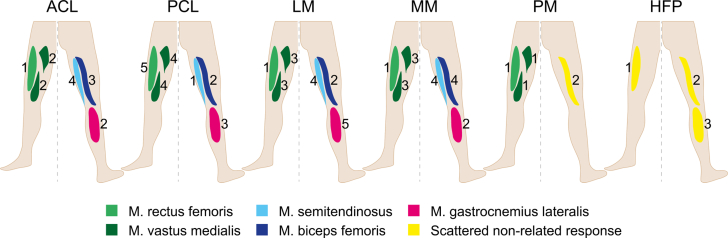
“MLR neuromapping”: Anatomical related representation of the mean medium MLR patterns after isolated stimulation of the ACL, PCL, LM, MM, HFP, and PM. The different colors indicate the activation of the respective muscle except yellow, which indicates a scattered non-related response. The numbers indicate the activation sequence of the muscles. (ACL, anterior cruciate ligament; HFP, Hoffa’s fat pad; LM, lateral meniscus; MLR, medium latency response; MM, medial meniscus; PCL, posterior cruciate ligament; PM, plica mediopatellaris.)

The neuromapping (Fig 5) combined the anatomical and quantitative representation of the individual muscular activation patterns after isolated mechanical stimulation of the ACL, PCL, LM, MM, HFP, and PM. The mean MLR was identified as the polysynaptic spinal reflex response and analyzed over time to establish the neuromapping. For the ACL, PCL, LM, and MM, a reproducible MLR was determined for all 5 muscles. Following isolated stimulation of the PM, only the M. vastus medialis displayed a reproducible reflex response. Stimulation of the HFP led to no reproducible MLR response.

## Discussion

The isolated intraoperative stimulation of the ACL and PCL, LM and MM, and PM resulted in distinct reproducible neuromuscular EMG responses of the thigh and shank muscles, corroborating our hypothesis. In detail, the direct neuromuscular connections between the PCL, LM, MM, the quadriceps, hamstring, and lateral gastrocnemius muscles were described for the first time. In addition, the PM, as part of the capsule, showed a direct connection to the m. vastus medialis. Although without clear reproducibility, patient-specific, independent muscle reflex responses could also be demonstrated for the HFP. The connection between the ACL and the thigh muscles was reestablished and extended by the direct reflex arc to the hamstring muscles.

### Latency Times

An overview of current literature values of latency times after stimulation of the cruciate ligaments and menisci is given in Table 3. The MLR after isolated ACL stimulation ranged from 43 to 79 milliseconds in the current study. The MLR of the hamstring muscles (48-79 milliseconds) followed those of the quadriceps musculature (43-61 milliseconds). These findings are in good agreement with those of Friemert et al.^29^ In their study, they investigated a similar patient cohort (n = 10) and initiated a mechanically induced neuromuscular response between the ACL and the hamstring muscles. They used an arthroscopic probe to transmit a mechanical stimulus of up to 300 N by means of a drop-weight impact to the ACL. The hamstring MLRs of the present study are slightly greater than those from Friemert et al. (40-50 milliseconds), which can be explained by a more rapid potential change of the hamstrings in the study of Friemert et al. resulting from a greater maximum force of 300 N compared with that of 40 N applied in the present study. Another more likely reason could be the different method of force application to the ACL. While we used an isolated direct impact by means of a customized impactor, Friemert et al. achieved the mechanical stimulus by transferring a tensile force by a dropped weight that was connected to a cable pull via a pulley that led to the ACL. Krogsgaard et al.^13^ reported hamstring MLR values of 60 to 120 milliseconds after ACL or PCL stimulation. While the values in the present study were slightly lower than those of Krogsgaard et al., they were still in the same range with 79 milliseconds for the connection between the hamstring muscles and the ACL and 55 milliseconds (range 38-85 milliseconds) for the hamstring–PCL connection. The antagonistic function of the ACL and PCL is reflected in the different activation patterns of the ventral and dorsal thigh muscles. Following ACL stimulation, there was first a change in potential of the quadriceps muscles followed by a change in potential of the hamstrings. The reverse activation pattern can be clearly observed in our quantitative (Fig 4) and qualitative (Fig 5) "neuromapping" after PCL stimulation. The latency times reported by Beard et al.^18^ are within our measurement range. However, it can be assumed that during a ventral tibial translation, the ACL was not stimulated in isolation. Therefore, the results are difficult to compare. Significantly longer MLR values after electrical stimulation were published by Dyhre-Poulsen and Krogsgaard^8^ and Fischer-Rasmussen et al.^9^ However, it remains unclear which receptors were electrically stimulated, although a stimulation via group II and III fibers of the mechanoreceptors is suspected. Nevertheless, it should be noted that pain stimuli are transmitted via group III fibers.^29^ As already described in 1967 by Freeman and Wyke,^34^ the ACL contains Golgi tendon organs, which have a conduction velocity in the axon of 75 m/s. The distance in a human of 185 cm from the knee joint to the lumbar connection height L3/4 and back to the middle of the thigh is approximately 120 cm. This leads to an arithmetical neural runtime of 16 milliseconds. Adding the multisynaptic connection with a delay of 1 millisecond per synapse and 3 milliseconds for the motor end plate, the reflex times measured in the present study are realistic. It further explains the MLR differences between the thigh and lower leg muscles with longer latencies of the M. gastrocnemius lateralis.

**Table 3:** Literature Overview of Medium Latency Response Values (MLR) in milliseconds, Resulting From Mechanical or Electrical Stimulation of Different Capsuloligamentous Structures on the Lower Leg Muscles

Structure	Author	Target Muscle and Related MLR Range
ACL	Friemert et al., 2005^29^	Hamstrings: 44 ± 4 ms
ACL	Friemert et al., 2005^28^	Hamstrings: 40-50 ms
ACL / PCL	Krogsgaard et al., 2002^13^	Hamstrings: 60-120 ms
ACL	Beard et al., 1994^7^	Hamstrings: 50-99 ms
ACL	Dyhre-Poulsen and Krogsgaard. 2000^8^	M. semitendinosus: 95 ± 35 ms∗
ACL	Tsuda et al., 2001^34^	Hamstrings: 50-80 ms∗
PCL	Fischer-Rasmussen et al., 2002^9^	M. quadriceps: 78-148 ms∗ Hamstrings: 88-110-ms∗ M. gastrocnemius: 189-258 ms∗
Menisci	Friemert et al., 2007^35^	Hamstrings: 39 ± 3 ms
ACL	Present study	M. vastus medialis: 49-61 ms M. rectus femoris: 43-58 ms M. semitendinosus: 58-79 ms M. biceps femoris: 48-68 ms M. gastrocnemius lateralis: 45-79 ms
PCL		M. vastus medialis: 44-76 ms M. rectus femoris: 61-85 ms M. semitendinosus: 38-51 ms M. biceps femoris: 41-55 ms M. gastrocnemius lateralis: 52-65 ms
LM		M. vastus medialis: 41-68 ms M. rectus femoris: 56-62 ms M. semitendinosus: 32-85 ms M. biceps femoris: 56-62 ms M. gastrocnemius lateralis: 56-74 ms
MM		M. vastus medialis: 49-67 ms M. rectus femoris: 40-61 ms M. semitendinosus: 33-83 ms M. biceps femoris: 55-78 ms M. gastrocnemius lateralis: 40-73 ms
PM		M. vastus medialis: 33-45 ms M. rectus femoris: 30-41 ms M. biceps femoris: 64-89 ms

ACL, anterior cruciate ligament; LM, lateral meniscus; MM, medial meniscus; PCL, posterior cruciate ligament; PM, plica mediopatelaris.

∗ MLR response after electrical stimulation—all other values result after mechanical stimulation.

Friemert et al.^35^ also determined mean values of 40 milliseconds for the MLR of menisci after ventral tibial translation. It can be assumed that not only a mechanical stimulus of the menisci but also of the ACL occurred during the translation maneuver. Therefore, a direct comparison of Friemert et al.’s results with the results of the present study is rather difficult.

Information about the knee joint capsule is only related to the histologic description of receptors.^36^ No neuromuscular, SLR, or MLR values have been reported to date. However, our findings indicated the shortest SLR values after the isolated mechanical stimulation of the PM. For the first time, we were able to perform an intraoperative detection of the reflex arc between the knee joint capsule and the thigh muscles, indicating latency times <40 milliseconds in the M. vastus medialis. Further individual SLR changes occurred in the M. rectus femoris and M. biceps femoris. These findings indicate that the joint capsule, in addition to its function as a passive stabilizer, plays an important role in the neuromuscular connectivity. Being the anatomically most superficial component of the capsular ligament structures, it surrounds the entire knee joint and changes its tension state with very little movement. Therefore, we assume that the short latency period until the potential change of the musculature can be considered an early protective mechanism to compensate for further stretching of the other internal knee joint structures. Therefore, care should be taken during arthroscopic interventions to ensure minimal compromising of the joint capsule.

Regarding the HFP, individual reflex responses were recorded without reproducibility. Interindividual polymorphisms of the neurohistologic structure of the knee joint capsule with variable density or complete absence of receptors could be an explanation for the differences between the patients. Fischer-Rasmussen et al.^9^ used an electrical stimulus of the HFP and found no electromyography potential change of the thigh or lower leg muscles.

The results of the present study help to improve the understanding of the neuromuscular relationship of the thigh and shank muscles with the capsuloligamentous knee joint structures. It can be concluded that the better we understand the neuromuscular regulation of the knee joint, the better we are able to understand both the surgical and physiotherapeutic treatment of capsuloligamentous knee joint structures.^37^ After a knee joint injury, it is important to restore both the mechanical and proprioceptive deficits of the capsuloligamentous structures of the patient. To prevent long-term consequences, including the development of early osteoarthritis, the neuromapping that was introduced in the present study might indicate strategies to optimize a combined and therefore, more efficient, treatment of the proprioceptive deficit. A postoperative treatment could be implemented in the form of neuromuscular training within the framework of physiotherapeutic exercises.^38^ One possible option for neuromuscular training in the immediate postoperative phase is the use of controlled active motion devices for patients after ACL or PCL reconstruction^39^ or neuromuscular electrical stimulation for neuromuscular rehabilitation.^40^ Therapeutic devices, for example, Aerostep, stepper, trampoline, bodyblade and the Posturomed, are useful in the rehabilitation process to train specific neuromuscular deficits. However, based on the results of the present study such exercises should not only be performed after ACL injuries,^41^ but also for postoperative rehabilitation after PCL, meniscus or joint capsule injuries.

### Limitations

One of the main limitations of the study is the low number of patients involved. Because of the different indications for the arthroscopic interventions, it was not possible to investigate all structures in each patient (ACL: n = 5; PCL: n = 4; LM: n = 7; MM: n = 8; PM: n = 6; HFP: n = 6). Therefore, the number of measurements per structure obtained in the present study is too low to allow an adequate statistical evaluation. Further, it remains to be examined what role individual reflex responses play after isolated mechanical stimulation of the HFP. A more reproducible HFP reflex outcome may be achievable with more patients and higher minimum values of the mechanical stimulation of the HFP. The latter can be determined, for example, using human specimens during pretests.

## Conclusions

Each stimulated structure displayed an individual MLR response, which allowed us to create neuromapping combining the anatomical and quantitative representations of the individual muscular activation patterns after isolated mechanical stimulation of the capsuloligamentous knee joint structures, corroborating our hypothesis.

## Supplementary Data

ICMJE author disclosure forms
